# Beneficial Effect of Fenofibrate and Silymarin on Hepatic Steatosis and Gene Expression of Lipogenic and Cytochrome P450 Enzymes in Non-Obese Hereditary Hypertriglyceridemic Rats

**DOI:** 10.3390/cimb44050129

**Published:** 2022-04-26

**Authors:** Rostislav Vecera, Martin Poruba, Martina Hüttl, Hana Malinska, Olena Oliyarnyk, Irena Markova, Zuzana Racova, Jan Soukop, Ludmila Kazdova

**Affiliations:** 1 Department of Pharmacology, Faculty of Medicine and Dentistry, Palacky University, 77515 Olomouc, Czech Republic; vecera@seznam.cz (R.V.); zuzu.matuskova@seznam.cz (Z.R.); jan.soukop01@upol.cz (J.S.); 2 Centre for Experimental Medicine, Institute for Clinical and Experimental Medicine, 14021 Prague, Czech Republic; martina.huttl@ikem.cz (M.H.); hana.malinska@ikem.cz (H.M.); ooliyarnyk@yahoo.com (O.O.); irena.markova@ikem.cz (I.M.); lukazdova@seznam.cz (L.K.)

**Keywords:** NAFLD, fenofibrate, silymarin, triglycerides, liver, CYP 2E1, CYP 4A1, lipoperoxidation

## Abstract

The efficacy of fenofibrate in the treatment of hepatic steatosis has not been clearly demonstrated. In this study, we investigated the effects of fenofibrate and silymarin, administered as monotherapy and in combination to existing hepatic steatosis in a unique strain of hereditary hypertriglyceridemic rats (HHTg), a non-obese model of metabolic syndrome. HHTg rats were fed a standard diet without or with fenofibrate (100 mg/kg b.wt./day) or with silymarin (1%) or with a combination of fenofibrate with silymarin for four weeks. Fenofibrate alone and in combination with silymarin decreased serum and liver triglycerides and cholesterol and increased HDL cholesterol. These effects were associated with the decreased gene expression of enzymes involved in lipid synthesis and transport, while enzymes of lipid conversion were upregulated. The combination treatment had a beneficial effect on the gene expression of hepatic cytochrome P450 (CYP) enzymes. The expression of the CYP2E1 enzyme, which is source of hepatic reactive oxygen species, was reduced. In addition, fenofibrate-induced increased CYP4A1 expression was decreased, suggesting a reduction in the pro-inflammatory effects of fenofibrate. These results show high efficacy and mechanisms of action of the combination of fenofibrate with silymarin in treating hepatic steatosis and indicate the possibility of protection against disorders in which oxidative stress and inflammation are involved.

## 1. Introduction

Non-alcoholic fatty liver disease (NAFLD) is the most common liver disease and is considered to be the hepatic manifestation of metabolic syndrome [1,2]. Lipid accumulation in the liver can be triggered by several mechanisms which include the increased hepatic uptake of circulating fatty acids, greater de novo fatty acid synthesis in hepatocytes, decreased hepatic beta oxidation, and lowered lipid transport from the liver [3]. This contributes to the fact that no specific drug is available for NAFLD treatment, but the association of NAFLD with dyslipidemia results in efforts for the condition to be treated with antihyperlipidemic drugs.

Fenofibrate (FF) is the most commonly used triglyceride-lowering drug. Its effects are mediated through transcription factor peroxisome proliferator-activated receptor alpha (PPARα) activation which regulates the expression of genes that encode enzymes involved in peroxisomal and mitochondrial β-oxidation, fatty acid transport, lipoprotein lipase activation, and triglyceride catabolism [4]. The result of the action of FF is the increased oxidation of triglycerides, their reduced secretion from the liver, and a decrease in blood levels [5].

Despite the abundance of studies regarding the efficacy of FF in reducing blood triglycerides, few have focused on the effects of fibrates on liver steatosis, and the results so far are controversial. In three placebo-controlled trials, fenofibrate did not reduce liver fat content in obese subjects or in patients with type 2 diabetes [6,7,8]. In another study, FF even increased total fat volume in the liver in patients with hypertriglyceridemia and NAFLD [9]. In animal experiments, FF mostly reduced hepatic steatosis [10], although increased liver lipids were also observed [11,12,13]. Moreover, sometimes, adverse side effects of FF therapy, such as liver injury, especially in the presence of high levels of human C-reactive protein (CRP), were observed [14,15].

Research efforts are therefore focused on finding additional substances with the ability to improve the efficiency of hypolipidemic therapy and/or mitigate its adverse effects. A suitable supplement to hypolipidemic treatment is silymarin (SLM), a standardized polyphenolic extract of milk thistle (Silybum marianum), which for its antioxidant, anti-inflammatory, and antifibrotic actions is widely used for treating liver injuries of various origins [16,17]. In some animal and human studies, SLM therapy has been shown to slightly reduce plasma triglyceride and cholesterol levels and increase HDL cholesterol [18,19,20,21]. On the other hand, studies on the effects of SLM in cases of hepatic steatosis are rare.

In the current study, we tested the hypothesis that the hepatoprotective effects of SLM would increase the efficacy of FF in the treatment of genetically induced hepatic steatosis. To enhance the efficiency of SLM, we used a micronized form, which increases bioavailability by up to 85% [22]. Previously, we found that the micronized form of SLM has greater beneficial effects on increasing plasma HDL cholesterol levels and lowering triglycerides compared to the standard form of SLM [23]. The therapeutic effect of the combination of these two substances has not yet been tested.

We used a unique animal strain of non-obese hereditary hypertriglyceridemic (HHTg) rat for the study. These rats of Wistar origin exhibit most symptoms associated with metabolic syndrome in humans: hypertriglyceridemia, liver steatosis, ectopic lipid accumulation, tissue insulin resistance, hyperinsulinemia, higher serum C-reactive protein, and mildly elevated blood pressure. This rat strain is an accepted model of metabolic syndrome which is not associated with obesity [24,25,26]. The use of a non-obese rat strain for the NAFLD study is justified. While NAFLD was initially thought to be present only in obese adults, a meta-analysis performed last year showed that 19% of patients with NAFLD were lean and 40% were non-obese [27]. In addition, the development of hepatic steatosis in HHTg rats is not associated with excessive dietary fat intake or genetically induced excessive obesity.

The aim of the study was to investigate the effects of FF and SLM, administered as monotherapy and in combination, on hepatic steatosis associated with genetic hypertriglyceridemia and determine the responsible mechanisms using the expression of genes that encode for enzymes involved in hepatic lipid metabolism, ATP-binding cassette transporters, and some cytochrome P450 family enzymes involved in the development of oxidative stress and inflammation.

## 2. Materials and Methods

### 2.1. Animals and Diets

All animal experiments were carried out using adult male hereditary hypertriglyceridemic rats (HHTg), provided by the Institute for Clinical and Experimental Medicine. The rats were maintained in a 12 h/12 h light-dark cycle at a temperature of 22–25 °C with free access to food and water. The rats were fed a standard laboratory diet without supplementation (controls) or supplemented with micronized fenofibrate (Fenofix^®^; Ingers Industrial Solutions, Czech Republic) in a dose of 100 mg/kg b.wt./day (FF), supplemented with 1% of micronized silymarin (SLM) (Favea, Koprivnice, Czech Republic), or supplemented with micronized fenofibrate (100 mg/kg b.wt./day) in combination with 1% micronized silymarin (FF+SLM) for 4 weeks. The standard laboratory diet consisted of 23% protein, 43% starch, 7% fat, 5% fiber, and 1% vitamin and mineral mixture (Bonagro, Blazovice, Czech Republic). The fenofibrate dose was chosen according to the literature [28]. The amount of food consumed was monitored daily throughout the experiment and did not differ among groups. At the end of the study, the rats were decapitated in a postprandial state, and their serum and tissues were collected for biochemical analyses. All animal experiments were performed in accordance with the Animal Protection Law of the Czech Republic 501/2020, which follows the European Community Council recommendations 86/609/ECC for the use of laboratory animals. All experiments on laboratory animals were approved by the Ethics Committee of the Ministry of Health, Czech Republic.

### 2.2. Gene Expression Assay

The RNeasy Mini Kit (Qiagen, Valencia, CA, USA) was used to isolate total mRNA. The amount of 1 µg of mRNA was used for cDNA synthesis using a Transcriptor High Fidelity cDNA synthesis kit (F. Hoffmann-La Roche AG, Basel, Switzerland). For the determination, TaqMan Gene Expression Assays (Thermo Fisher Scientific, Waltham, MA, USA) containing target primers and a sequence-specific probe optimized for the best functional performance were used. Commercially available primers purchased from the same corporation were used to determine the mRNA of lipoprotein lipase (*Lpl*), fatty acid synthase (*Fas*), 3-hydroxy-3methyl-glutaryl-coenzyme A reductase (*Hmgcr*), ATP-binding cassette transporter sub-family b member 1(*Abcb1a* and *Abcb1b*), and cytochrome P450 (*Cyp7a1*, *Cyp4a1*, and *Cyp2e1*). The real-time PCR was performed on 1536-well plates using acoustic liquid handler Echo 550 (Labcyte, Dublin, Ireland) and LightCycler 1536 Instrument (F. Hoffmann-La Roche AG, Basel, Switzerland). Measurements were performed in parallels of six reverse transcription, and quantitative real-time PCR analyses for the expression of the *Scd1* gene in the liver were performed using the TaqMan RNA-to-CTTM 1-Step Kit and the TaqMan Gene Expression Assay (Applied Biosystems, Foster City, CA, USA) and carried out in a Vii ATM 7 Real-Time PCR System (Applied Biosystems, Foster City, CA, USA). The results were evaluated using the “Delta-Delta Ct” method, and the data were normalized and related to the *Hprt* gene (Thermo Fisher Scientific, Waltham, MA, USA) or *Pgk1* gene (for the *Scd1* gene).

### 2.3. Biochemical Analysis

Serum concentrations of triglycerides and total cholesterol were measured using commercially available kits (Erba-Lachema, Brno, Czech Republic). HDL cholesterol was measured after precipitation with dextran and MgCl2 via the spectrophotometric method using a kit (Roche Diagnostics GmbH, Mannheim, Germany). For the measurement of liver triglycerides and cholesterol concentration, tissues were powdered and extracted in chloroform:methanol (2:1). After extraction, the organic phase was collected and evaporated under nitrogen gas. Extracted lipids were dissolved in isopropyl alcohol, and the concentration of triglycerides and cholesterol was measured using enzymatic assays (Erba-Lachema, Brno, Czech Republic). The parameters of oxidative stress were assessed according to the concentrations of lipoperoxidation products. Liver-conjugated dienes (CDs) were analyzed in lipid extracts and measured spectrophotometrically according to the method described previously [29]. The final products of lipoperoxidation were analyzed spectrophotometrically using the reaction with thiobarbituric acid and expressed as thiobarbituric acid-reactive substances (TBARS) adjusted to the tissue protein concentration [15].

### 2.4. Statistical Analysis

The normality of the data was tested using the Shapiro–Wilk test and analyzed using ANOVA and post hoc Bonferroni correction using Statistica software (ver. 12, Statsoft CZ, Prague, Czech Republic). Statistical significance was defined as *p* ≤ 0.05.

## 3. Results

### 3.1. Effects of FF and SLM on Serum and Liver Lipids

Although food intake did not differ between groups (data not shown), the body weights of rats treated with FF without or with added SM were lower by 11% and 7% (both *p* < 0.01), respectively, in comparison with the control group or animals treated with SLM alone. (Table 1). The decrease in body weight after FF treatment is well known and is explained by the increased oxidation of fatty acids, especially in muscle tissue.

As shown in Table 1, FF alone and similarly in combination with SLM markedly reduced the serum concentration of triglycerides (both *p* < 0.001) and the concentrations of serum total cholesterol by 36% (both *p* < 0.001) compared to the untreated controls. The administration of SLM alone reduced serum triglycerides levels significantly (−19%; *p* < 0.01), but serum total cholesterol levels did not differ compared to the untreated controls. A remarkable finding was an increase in serum HDL cholesterol levels after the administration of FF alone (+49%; *p* < 0.05) and in combination with SLM (+44%; *p* < 0.01). Treatment with SLM alone also increased serum HDL cholesterol levels by +49% (*p* < 0.05) compared to the untreated controls.

Hepatic triglyceride concentrations were significantly reduced in FF- (−67%; *p* < 0.001) and FF+SLM-treated (−65%; *p* < 0.001) rats in comparison to the untreated controls. Both FF- and FF+SLM-treated rats exhibited decreased concentrations of hepatic cholesterol (both *p* < 0.01) compared to untreated or SLM-treated animals. In contrast, the administration of SLM alone did not reduce the accumulation of triglycerides or cholesterol in the liver (Table 1).

Table 1 shows that in the liver, FF treatment led to an increased production of initial lipoperoxidation products-conjugated dienes (CDs) by 21% (nonsignificant) and final lipoperoxidation products—TBARS (thiobarbituric acid reactive substances)—by 27% (*p* < 0.01). In contrast, SLM monotherapy favorably affected lipoperoxidation, as evidenced by a 25% decrease in CD and a 29% decrease in TBARS (both *p* < 0.05) compared to the control group. The beneficial effect of SLM was also manifested in combination with FF, as shown by the reduction in TBARS concentrations (−36% + *p* < 0.01) in the FF + SLM-treated group compared to FF alone (Table 1).

### 3.2. Effects of FF and SLM on Gene Expression

**Figure 1:**
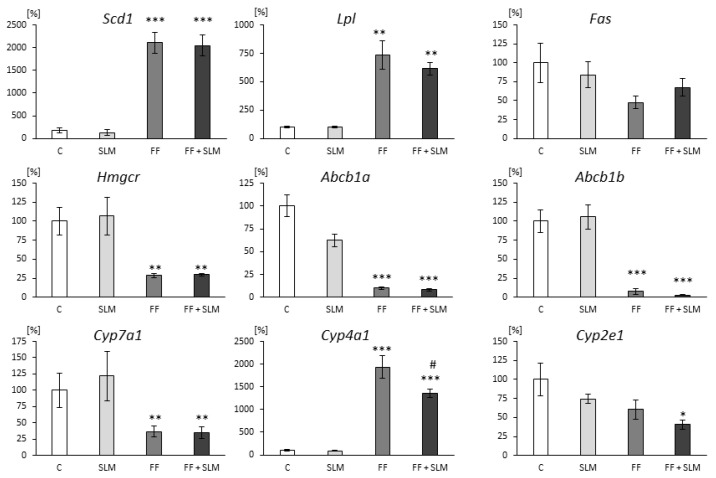
Hepatic expression of genes involved in lipid metabolism, drug transport proteins, and the cytochrome P450 system in HHTg rats treated with fenofibrate (FF), silymarin (SLM), and combination of SLM+FF compared to untreated HHTg control rats (C). Data are expressed as mean ± SEM; *n* = 6–7 animals per group. Significantly different from untreated controls: * *p* < 0.05, ** *p* < 0.01, *** *p* < 0.001; significantly different from FF diet: # *p* < 0.05.

To search for the mechanisms responsible for the effects of the tested substances, we analyzed the expression of genes encoding enzymes involved in hepatic lipid metabolism, ATP-binding cassette transporters, and cytochrome P450 family enzymes. The treatment with FF alone and in combination with SLM was associated with an excessive increase in hepatic stearoyl-CoA desaturase 1 (Scd 1) expression (Figure 1). This gene encodes the enzyme (SCD1) responsible for the synthesis of monounsaturated fatty acids (MUFAs). Compared to the untreated group, FF and FF+SLM treatment caused considerable increases in *Lpl* mRNA expression (by 635% for FF and 514% for FF+SLM; both *p* < 0.01) that encodes lipoprotein lipase, a rate-limiting enzyme for plasma triglyceride clearance and the tissue uptake of fatty acids (Figure 1). Hepatic fatty acid synthase (FAS) catalyzes the de novo synthesis of fatty acids. Figure 1 shows a slight decrease in *Fas* mRNA expression in FF alone (−53%) and in FF with combination with the SLM-treated (−33%) group. The lower accumulation of cholesterol in the liver after FF and FF+SLM treatment was associated with lower mRNA expression of the *Hmgcr* gene (by 70%; *p* < 0.01) coding HMGCR, a key enzyme involved in cholesterol synthesis (Figure 1). The administration of SLM alone did not affect hepatic *Scd1*, *Lpl*, *Fas* and *Hmgcr* gene expression compared to the control group (Figure 1).

Furthermore, we analyzed the gene expression of the hepatic cholesterol and drugs transporters *Abcb1a* (Figure 1) and *Abcb1b* (Figure 1) encoding P-glycoprotein (P-gp). The hepatic mRNA expression of transporters was significantly reduced in FF- and FF+SLM-treated rats (both *p* < 0.0001) in comparison with the untreated group. On the other hand, SLM alone did not affect the expression of these transporters. Data concerning the effect of FF alone on P-gp were published previously [30].

Next, we analyzed the effect of FF and SLM on the hepatic mRNA expression of selected cytochrome P450 family enzymes involved in lipid metabolism.

Gene *Cyp7a1* encodes the enzyme cholesterol 7α-hydrolase which is the rate-limiting enzyme responsible for the synthesis of bile acids and thus the excretion of cholesterol from the body. As shown in Figure 1, *Cyp7a1* expression was significantly decreased in the group treated with FF (by 63%; *p* < 0.01), and FF+SLM (by 65%; *p* < 0.01) compared to the untreated group. SLM alone did not affect this expression. The hepatic CYP4A1 enzyme plays an important role in the metabolism of fatty acids by ω-hydroxylation. We observed the increased mRNA expression of *Cyp4a1* after FF treatment by 1837% and a significant decrease in FF + SLM-treated rats (−37%), (Figure 1). There were no significant differences in the expression of *Cyp4a1* between SLM monotherapy and untreated control animals (Figure 1).

The enzyme CYP2E1 catalyzes the biotransformation of many endogenous substrates such as fatty acid and ketone bodies and, in contrast to other cytochrome P450 enzymes, can generate excessive amounts of reactive oxygen species (ROS) during the oxidation of fatty acids. As shown in Figure 1, the hepatic gene expression of *Cyp2e1* was slightly (non-significant) reduced in SLM and FF monotherapy, while the co-administration of SLM + FF reduced the expression by 59% (*p* < 0.05) compared to untreated animals.

## 4. Discussion

The accumulation of lipids in the liver leads to the progression of liver damage and increases the risk of developing metabolic syndrome, type 2 diabetes, and cardiovascular and chronic kidney disease [1]. This focuses attention on the liver as a main target to treat these metabolic disorders, but so far, no effective therapy for NAFLD has been found. In the current study, we tested the effects of FF and SLM, administered as monotherapy and in combination, on already-developed hepatic steatosis in non-obese hereditary hypertriglyceridemic rats that exhibited other disorders associated with the metabolic syndrome [24,25,26]. In addition, this model is not associated with excessive obesity or excessive fat intake as in most other models. Our results showed that in HHTg rats, FF lowered serum triglyceride and cholesterol levels, increased HDL cholesterol levels, and reduced liver steatosis with high efficacy. We found that the mechanism of these beneficial effects may involve changes in the gene expression of *Lpl*, *Fas*, *Scd1*, and *Hmgcr*, and transport proteins *Abcb1a*, *Abcb1b*, and *Cyp7a1*. A negative consequence of FF therapy was the increased production of lipoperoxidation products in the liver, as evidenced by increased concentrations of conjugated dienes and TBARS and the up-regulation of hepatic Cyp4a gene expression, involved in the production of pro-inflammatory mediators.

The addition of SLM to FF therapy did not change the beneficial hypolipidemic effect of FF but downregulated the hepatic gene expression of *Cyp2e1* and FF-induced overexpression of *Cyp4a1*, which indicates possible beneficial effects on the formation of inflammatory mediators and pro-oxidation products. The amelioration of the negative effect of FF on lipoperoxidation was consistent with our findings. To our knowledge, this is the first study to monitor the effect of FF on hepatic steatosis associated with genetic hypertriglyceridemia without the presence of inappropriate dietary fat or excessive obesity. It is also the first study to focus on the effects of FF therapy in combination with hepatoprotective silymarin. Possible molecular mechanisms responsible for the effects of these substances are discussed below.

### 4.1. Effects of FF Monotherapy

The lipid-modifying activity of fibrates is mediated by the activation of the nuclear receptor PPARα that affects intracellular lipid metabolism through the direct transcriptional control of genes involved in fatty acid transport, mitochondrial and peroxisomal β-oxidation, lipoprotein lipase activation, and triglyceride and cholesterol synthesis and catabolism [31,32]. To investigate the mechanisms responsible for the hypolipidemic effects of FF, we analyzed the hepatic mRNA expression of genes involved in lipid and cholesterol metabolism.

A somewhat unexpected finding was a high increase in hepatic *Scd1* gene expression in FF-treated hypertriglyceridemic rats. The *Scd1* gene codes for SCD1, an enzyme necessary for conversion of saturated fatty acid to monosaturated fatty acid (MUFA) production that serves as a substrate for the synthesis of triglycerides, phospholipids, cholesteryl esters, and other metabolic products [33]. The role of the SCD1 in the development of NAFLD is still not fully explained, and the increased expression of *Scd1* may be an adaptive response to the increased catabolism of triglycerides by FF-induced increased β-oxidation. Oosterveer et al., using a stable isotope technique method, provided evidence that FF treatment increased hepatic fatty acid synthesis and their chain elongation to MUFA as protection against fatty acid cytotoxicity [34]

Our results showed that FF treatment increased the gene expression of *Lpl* in the liver five- to six-fold compared to the untreated group. As the enzyme LPL is rate limiting for plasma triglyceride clearance and the tissue uptake of fatty acids, its activity is carefully controlled to ensure the supply of fatty acids to tissue oxidation (muscle and brown adipose tissue), storage (white adipocytes) or the formation of lipid metabolites (liver). LPL activity is differentially regulated in various tissue; in the liver and muscles, predominantly by PPARα [35]. An FF-induced increase in *Lpl* expression in HHTg rats suggests that the beneficial effects of FF on circulating lipids and hepatic steatosis can be mediated by PPARα-activated LPL. In contrast, FF treatment did not affect the release of fatty acids from adipose tissue (results not shown), suggesting that PPARγ is not involved in the effects of FF.

Another enzyme that could be targeted for the treatment of hepatic steatosis is FAS, which contributes to the de novo synthesis of lipids for storage and secretion. Our results showing a slight and insignificant decrease in FAS mRNA expression in FF-treated rats suggest that this enzyme is less involved in the mechanism of the hypolipidemic effect of FF on hepatic steatosis. This is consistent with the observations in the liver-specific FAS knockout mice that the contribution of FAS to stored triglycerides is only around 11% [36].

The cholesterol-lowering effects of FF observed in liver of HHTg rats were associated with the reduced hepatic gene expression of *Hmgcr*, encoding a key enzyme for the synthesis of cholesterol. Additionally, the mRNA expression of the *Abcb1a* and *Abcb1b* genes was markedly reduced after FF treatment. These genes encode rat membrane protein P-glycoprotein (P-gp) involved not only in the efflux of cytotoxic substances from cells but also in intracellular cholesterol transport and lipid homeostasis [34,37]. So far, the inhibitory effect of FF on P-gp has only been demonstrated in vitro on a cell line expressing human P-gp [38] and in the liver of HHTg rats treated with FF [30].

Another way to reduce the concentration of cholesterol in the liver is its elimination as bile acids.

The hepatic CYP7A1 is a rate-limiting enzyme responsible for cholesterol conversion to 7a-hydroxycholesterol, which is then secreted into bile acids and eliminated from an organism [39]. We found reduced hepatic *Cyp7a1* expression in FF-treated HHTg rats. Relatively few studies have addressed the effect of FF on *Cyp7a1* expression to date, and the results of the studies are equivocal. As in our study, FF administration reduced hepatic *Cyp7a1* expression in clinical trials [40,41]. In contrast, in animal models using a high-fat diet or obese strains of mice and rats to induce NAFLD, FF increased *Cyp7a1* expression [42,43]. These results indicate that the effects of FF on *Cyp7a1* expression may vary depending on the model studied, including the amount of cholesterol in the diet.

Our results showed that FF led to the upregulation of *Cyp4a1* gene expression in HHTg rats in comparison to the untreated control. *Cyp4A* isoforms catalyze the microsomal omega-hydroxylation of fatty acids that convert arachidonic acid to 19- and 20-hydroxyeicosatetraenoic acids (HETEs), exhibiting a proinflammatory function. We have previously found a proinflammatory effect of fibrates mediated by the increased hepatic expression of *Cyp4A* in cholesterol-fed Wistar rats [44]. The increased hepatic expression of *Cyp4a* was observed in obese Zucker rats treated with ciprofibrate [45]. The pro-inflammatory activity of FF could contribute to the hepatotoxic effects of FF, which we found in spontaneously hypertensive rats (SHRs) expressing human C-reactive protein. SHR transgenic rats (SHR-CRPs) treated with FF in comparison with SHR-CRP untreated controls showed increased serum levels of proinflammatory marker IL-6 (interleukin 6), MCP-1(monocyte chemoattractant protein 1), increased concentrations of AST and ALT, and increased oxidative stress and necrotic changes in the liver [15].

Increased CYP4A1 expression in FF-treated HHTg rats together with these findings indicates possible negative effects of FF. Another negative consequence of FF treatment was the increased production of lipoperoxidation products in the liver which may increase the risk of progression of liver steatosis to more severe liver disease. These data suggest the need to search for additional substances that would mitigate these negative effects and improve the effects of hypolipidemic therapy in tissues.

### 4.2. Effect of SLM Monotherapy

SLM is commonly used as a hepatoprotective drug in different types of liver disorders for its anti-oxidant, anti-inflammatory and anti-fibrotic actions [46,47]. Our results showed that SLM treatment slightly reduced circulating triglyceride levels (−19%) and increased HDL cholesterol levels (+49%) without affecting the hepatic steatosis. There is plentiful evidence showing that circulating HDLs protect low density lipoprotein (LDL) particles from oxidative damage by free radicals, resulting in the inhibition of pro-inflammatory oxidized lipids generation and the lower progression of liver injury and reduced affinity to macrophage receptors and the development of cardiovascular complications [48]. The beneficial effects of SLM on dyslipidemia are consistent with our previous studies [19,23]. Recently, the lipid-lowering effect of SLM was demonstrated in two meta-analyses of clinical trials [21,49] and in one study on patients with type 2 diabetes [20]. The mechanisms of the hypolipidemic effect of SLM observed in circulation are not well-understood. In our experiment, SLM treatment alone did not affect the gene expression of liver enzymes involved in lipid synthesis, lipid transport, or cytochrome P450 enzymes participating in the oxidation and hydroxylation of fatty acids. The only exception was a slight decrease in CYP2E1 expression, which, however, was not statistically significant. A significant effect of SLM monotherapy was the reduced production of lipoperoxidation products, conjugated dienes, and TBARS in the liver. The mechanism of these actions may be related to its well-known antioxidant effects such as a free radical scavenging or influence on the enzyme systems associated with antioxidant defense [17,50].

### 4.3. Effect of Combination Therapy FF with SLM

In this study, the results show the beneficial effects of FF on blood lipids and the amelioration of hepatic steatosis associated with genetically conditioned hypertriglyceridemia. However, long-term treatment with FF may cause liver dysfunction [14,51,52]. To improve hypolipidemic therapy and avoid negative side effects, we investigated the effect of combination therapy of FF with hepatoprotective SLM. The combined treatment with FF+SLM did not change the hypolipidemic effects of FF administered alone nor had any additional effect on elevated blood HDL cholesterol levels. Similarly, the FF-induced reduction in triglyceride and cholesterol accumulation in the liver was not further affected by the combination of FF with SLM. Similarly, the gene expression of enzymes involved in lipid synthesis and transport was not altered when SLM was added to FF treatment. However, our results also showed the new and important beneficial effects of adding SLM to FF therapy. This combination significantly reduced the mRNA expression of the *Cyp4a1* isoform which encodes enzymes catalyzing the conversion of arachidonic acids to pro-inflammatory eicosanoids [53] and whose expression was markedly stimulated by the administration of FF alone.

An important observation in this study was the effect of the combination of FF+SLM, which caused a significant decrease in hepatic *Cyp2e1* expression. The role of this enzyme in the pathogenesis of NAFLD is under active investigation for its prominent role in the progression of hepatic damage. In contrast to other cytochrome P450s, CYP2E1 is able to produce significant amounts of ROS. CYP2E1 plays a role in the metabolism of endogenous compounds such as fatty acids, ketone bodies, and glycerol and also catalyzes the oxidation of a variety of small molecules including drugs and alcohol in which ROS are generated and which subsequently induce oxidative stress and cause damage to cellular components [53,54,55,56]. This may contribute to the progression of simple hepatic steatosis to more severe disorders, including fibrosis and steatohepatitis [57]. The search for CYP2E1 inhibitors is therefore very important for the effective treatment of NAFLD, and our results suggest that the combination of FF with SLM could be such a treatment. This assumption is also supported by our results showing that the combination of FF with SLM significantly reduced FF-induced increases in the formation of lipoperoxidation products in the liver.

## 5. Conclusions

Our results show that FF effectively reduced hepatic steatosis induced by genetically determined hypertriglyceridemia. The mechanisms through which FF modulated triglycerides and cholesterol accumulation in the liver involves the gene expression of enzymes regulating lipid synthesis, transport, and some cytochrome P450-regulating metabolic transformations. The combined administration of FF with SLM did not change the beneficial hypolipidemic effects of FF, and in addition, reduced lipoperoxidation, the gene expression of CYP4A1 and CYP2E1 involved in the reduction in pro-inflammatory activity, and the production of ROS, and thus decreased the risk of developing complications of hepatic steatosis. These data provide important new findings on possible improvements in the therapy of hepatic steatosis associated with hypertriglyceridemia and the metabolic syndrome.

## Figures and Tables

**Table 1:** Body weight and metabolic parameters in HHTg rats treated with silymarin, fenofibrate, and their combination.

Parameter	C	SLM	FF	FF + SLM
Body weight (g)	414 ± 4	406 ± 2	370 ± 7 ^b^ **	386 ± 7 ^c^ **
Serum lipids				
Triglycerides (mmol/L)	4.95 ± 0.22	4.03 ± 0.12 ^a^ **	1.15 ± 0.04 ^b^ ***	1.16 ± 0.55 ^c^ ***
Cholesterol (mmol/L)	1.59 ± 0.05	1.60 ± 0.03	1.01 ± 0.03 ^b^ ***	1.00 ± 0.03 ^c^ ***
HDL cholesterol (mmol/L)	0.43 ± 0.05	0.62 ± 0.06 ^a^ *	0.62 ± 0.03 ^b^ *	0.62 ± 0.03 ^c^ **
Liver lipids				
Triglycerides (μmol/g)	8.92 ± 0.66	7.57 ± 0.46	2.80 ± 0.10 ^b^ ***	3.13 ± 0.22 ^c^ ***
Cholesterol (μmol/g)	6.55 ± 0.18	6.57 ± 0.17	4.83 ± 0.20 ^b^ **	4.87 ± 0.18 ^c^ **
Lipoperoxidation products in the liver				
CD (nmol/mg proteins)	31.5 ± 2.4	23.8 ± 2.0 ^a^ *	38.0 ± 2.7	38.4 ± 2.2
TBARS (nmol/mg proteins)	1.69 ± 0.18	1.21 ± 0.12 ^a^ *	2.14 ± 0.15 ^b^ *	1.37 ± 0.12 ^d^ **

Data are expressed as means ± SEM; *n* = 6–7 animals per group. Abbreviations: C—untreated controls; SLM—silymarin; FF—fenofibrate; FF+SLM—fenofibrate + silymarin; CD—conjugated dienes; TBARS—thiobarbituric acid-reactive substances. Statistically significant differences: denotes * *p* < 0.05; ** *p* < 0.01; *** *p* < 0.001 for ^a^ control versus SLM; ^b^ control versus FF; ^c^ control versus FF + SLM; ^d^ FF vs. SLM.

## Data Availability

All datasets generated for this study are included in the article.

## Abbreviations

ABCB1a, ABCB1b	ATP-binding cassette transporter sub-family b member 1
ALT	alanine aminotransferase
AST	aspartate aminotransferase
CYP7A1, CYP4A1, CYP2E1	cytochrome P450
Fas	fatty acid synthase
FF	fenofibrate
HHTg	hereditary hypertriglyceridemic
HMGCR	3-hydroxy-3methyl-glutaryl-coenzyme A reductase
IL-6	interleukin 6
LPL	lipoprotein lipase
MCP-1	monocyte chemoattractant protein 1
MUFA	monounsaturated fatty acids
NAFLD	nonalcoholic fatty liver disease
PPARα	peroxisome proliferator-activated receptor alpha
ROS	reactive oxygen species
SCD1	stearoyl-CoA desaturase
SHR	spontaneously hypertensive rats
SLM	silymarin
